# Effect of allergens and irritants on levels of natural moisturizing factor and corneocyte morphology

**DOI:** 10.1111/cod.12770

**Published:** 2017-03-14

**Authors:** Sjors A. Koppes, Suzana Ljubojević Hadžavdić, Ivone Jakasa, Nika Franceschi, Christoph Riethmüller, Ružica Jurakić Tončic, Branka Marinovic, Nidhin Raj, Anthony V. Rawlings, Rainer Voegeli, Majella E. Lane, Marek Haftek, Monique H.W. Frings‐Dresen, Thomas Rustemeyer, Sanja Kezic

**Affiliations:** ^1^ Academic Medical Center, Department: Coronel institute of Occupational Health Amsterdam Public Health Research Institute 1105 AZ Amsterdam The Netherlands; ^2^ Department of Dermatology‐Allergology VU University Medical Centre 1081 HV Amsterdam The Netherlands; ^3^ Department of Dermatology and Venereology University Hospital Centre Zagreb and University of Zagreb School of Medicine 10000 Zagreb Croatia; ^4^ Faculty of Food Technology and Biotechnology, Laboratory for Analytical Chemistry, Department of Chemistry and Biochemistry University of Zagreb 10000 Zagreb Croatia; ^5^ Department of Dermatology and Venereology, University Clinical Hospital Centre ‘Sestre Milosrdnice’ 10000 Zagreb Croatia; ^6^ Centre for Nanotechnology, Serend‐ip GmbH Centre for Nanotechnology 48149 Münster Germany; ^7^ Skin Research Laboratory, Department of Pharmaceutics University College London WC1N 1AX London UK; ^8^ DSM Nutritional Products Ltd. 4002 Basel Switzerland; ^9^ Laboratory of Tissue Biology and Therapeutic Engineering CNRS and University of Lyon UMR 5305 Lyon France

**Keywords:** allergic contact dermatitis, Dermal Texture Index, irritant contact dermatitis, natural moisturizing factor

## Abstract

**Background:**

The irritant sodium lauryl sulfate (SLS) is known to cause a decrease in the stratum corneum level of natural moisturizing factor (NMF), which in itself is associated with changes in corneocyte surface topography.

**Objective:**

To explore this phenomenon in allergic contact dermatitis.

**Methods:**

Patch testing was performed on patients with previously positive patch test reactions to potassium dichromate (Cr), nickel sulfate (Ni), methylchloroisothiazolinone (MCI)/methylisothiazolinone (MI), or p‐phenylenediamine. Moreover, a control (pet.) patch and an irritant (SLS) patch were applied. After 3 days, the stratum corneum from tested sites was collected, and NMF levels and corneocyte morphology, expressed as the amount of circular nanosize objects, quantified according to the Dermal Texture Index (DTI), were determined.

**Results:**

Among allergens, only MCI/MI reduced NMF levels significantly, as did SLS. Furthermore, only MCI/MI caused remarkable changes at the microscopic level; the corneocytes were hexagonal‐shaped with pronounced cell borders and a smoother surface. The DTI was increased after SLS exposure but not after allergen exposure.

**Conclusions:**

MCI/MI significantly decreased NMF levels, similarly to SLS. The altered corneocyte morphology suggests that skin barrier damage plays a role in the pathogenesis of MCI/MI contact allergy. The DTI seems to differentiate reactions to SLS from those to the allergens tested, as SLS was the only agent that caused a DTI increase.

Allergic contact dermatitis (ACD) and irritant contact dermatitis (ICD) are common inflammatory skin diseases that pose a major problem in public health because of the widespread use of skin irritants and/or contact allergens in occupational settings and in consumer products. Although these two forms of contact dermatitis have different pathogenesis, they show similar clinical features, including erythema, fissuring, and vesicles, and, in a more severe form, bullae 1, 2. The primary step in the development of ICD is, for most skin irritants, characterized by disruption of the skin barrier, which is followed by activation of the innate immune system without involvement of T cells 3. A genetic deficiency of the epidermal protein profilaggrin is a strong predisposing factor for ICD 4, 5. The odds ratio (OR) for filaggrin gene (*FLG*) mutations, adjusted for atopic dermatitis (AD), was 1.61, whereas individuals with a history of AD who are also carriers of an *FLG* mutation have a 4.7‐fold risk for ICD 6. A history of AD increases the risk for ICD threefold. It has to be noted, however, that AD patients without *FLG* mutations also have reduced filaggrin expression caused by T helper 2‐mediated inflammation in AD 7. Filaggrin and its degradation products, which are the main constituents of natural moisturizing factor (NMF), are responsible for a number of functions concerning skin barrier function in the stratum corneum, including mechanical properties, skin hydration, and the epidermal inflammatory response 8. Recently, it has been shown that various skin irritants significantly reduce the levels of NMF 3. NMF levels, in turn, showed a strong association with corneocyte surface morphology, expressed as the Dermal Texture Index (DTI), supporting the view that alterations in the skin barrier play a major role in ICD 9.

The effect of contact allergens on the skin barrier has not been extensively studied to date, and, if so, it has been mainly assessed with skin bioengineering techniques such as transepidermal water loss measurement 10. However, skin barrier defects arising from concomitant irritant properties of an allergen may play an important role in the activation of the adaptive immune response and the development of ACD 10, 11, 12, 13. ACD is a type IV cell‐mediated immune reaction, separated into two distinct phases: the sensitization phase, in which the immune system is primed to react to a given allergen [usually molecules with a molecular weight (MW) of <500], and the elicitation phase, following re‐exposure. In this process, the impaired skin barrier may facilitate sensitization in the first place, but also the allergic response as a result of the increased penetration of contact allergens 8, 11, 12. The structural components of the stratum corneum, such as the extracellular lipid matrix, the cornified envelope, and the corneodesmosomes, are primary targets for most irritants 13. Recently, it has been shown that the model irritant sodium lauryl sulfate (SLS) affects the expression of filaggrin 14, 15. Similar morphological changes have also been seen in mice when they are exposed to 2,4,6‐trinitro‐1‐chlorobenzene, a potent contact allergen 16. Studies on the effect of contact allergens on corneocyte morphology in humans are lacking. Therefore, in the present study, we investigated the levels of NMF, the associated filaggrin‐degradation enzymes bleomycin hydrolase (BH) and calpain‐1 (C‐1) and stratum corneum plasmin, as an indicator of skin barrier damage and corneocyte surface topography and morphology 9, 17, 18. We focused on the effects of skin exposure to clinically relevant allergens: potassium dichromate (Cr), nickel sulfate (Ni), methylchloroisothiazolinone (MCI)/methylisothiazolinone (MI), and *p*‐phenylenediamine (PPD), and to the model irritant SLS.

## Methods

### Patients

The database of the dermatological outpatient clinic of the Zagreb University Hospital was screened for individuals with positive patch reactions, clinically graded according to the ESCD/ICDRG guidelines as 1+ or 2+, to one of four common contact allergens: Cr, Ni, PPD, and MCI/MI 19. Patients with two or more 1+ or 2+ reactions to Cr, Ni, PPD or MCI/MI were preferred, as multiple allergens could be tested in 1 individual. Patients with a 3+ reaction were not selected, to avoid severe reactions that might impair tape stripping. Patients with a history of AD were excluded. The experimental protocol followed the Declaration of Helsinki Principles, and was approved by the Medical Ethics Committee of the University Hospital Centre Zagreb. Written informed consent was obtained from each participant.

### Procedure

All participants were patch tested on the back with one or two allergens to which the participant had previously shown a 1+ or 2+ patch test reaction, and also with SLS and pet. The tested substances were applied in van der Bend chambers (van der Bend, Brielle, The Netherlands), namely, PPD 1% pet., potassium dichromate 0.5% pet. (Almirall Hermal, Reinbeck, Germany), nickel sulfate 5% pet., and MCI/MI 3:1 in 0.01% aq. (Smartpractice Europe, Barsbüttel, Germany). To provoke ICD, patches with 1% and 2% SLS aq. were used. A patch with the vehicle (100% pet.) was used as a control. Four identical patch series were applied: two series on the left and two series on right side of the upper back. Two identical series on the left side were used for respective day (D) 2 and D3 assessment, enabling stripping on ‘fresh’ non‐stripped skin sites. The patches on the right side of the back functioned as a back‐up for possible technical failures. On D2, all patches were removed, patch sites were marked, and the skin was allowed to rest for 30 min. On D2 and D3, respectively, the stratum corneum samples from the skin sites where the duplicate patches had been applied were collected with adhesive tape (1.5 cm^2^, D‐Squame; CuDerm, Dallas, TX, USA) 20. In total, eight consecutive tape strips were taken from each patch application site for analysis. Different tapes were used for the various analyses; tape 3 was used for atomic force microscopy (AFM), tape 4 for proteases, and scanning electron microscopy (SEM), and tape 5 for NMF analysis.

### NMF and protease activity

NMF was defined as the sum of the concentrations of pyrrolidone carboxylic acid, urocanic acid, and histidine. NMF levels were determined according to a method described in detail elsewhere 21. Briefly, the fifth tape strip was extracted with 0.5 ml of 25% ammonia. The ammonia extract was evaporated and the residue was dissolved in 250 µl of water before analysis by high‐performance liquid chromatography (HPLC). The NMF level was normalized for the stratum corneum protein amount determined with a Pierce Micro BCA protein assay kit (Thermo Fisher Scientific, Rockford, IL, USA) to compensate for the variable amount of the stratum corneum protein on the tape strips. Enzymatic activities of BH, C‐1 and plasmin were determined in eight randomly selected subjects who were positive either for Ni or MCI/MI and their corresponding unpatched and pet. test sites. The analysis has previously been described in detail by Raj et al. and Voegeli et al. 17, 22, 23, 24, 25. Briefly, buffer extracts of the tape strips (250 µl) were combined with fluorogenic peptide substrates (1.25 µl) (for BH‐like activity, H‐Cit‐AMC; for C‐1‐like activity, Suc‐Leu‐Leu‐Val‐Tyr‐AMC; and for plasmin‐like activity, MeOSuc‐Ala‐Phe‐Lys‐AMC), and agitated at 1000 rpm at 37°C. The reaction was stopped after 2 h by adding acetic acid (250 µl). The released AMC was quantified by reverse‐phase HPLC (excitation at 354 nm; emission at 442 nm), and the results were corrected for stratum corneum protein content on the tape strips as determined with the 850‐nm absorption infrared densitometer SquameScan 850A (Heiland Electronic, Wetzlar, Germany), according to a procedure described elsewhere 24.

### Corneocyte morphology

Corneocytes from patients were analysed by AFM as described by Franz et al. 26. Briefly, the third consecutive tape strip was subjected to AFM measurements carried out with a Multimode atomic force microscope equipped with a Nanoscope III controller and software version 5.30sr3 (Digital Instruments, Santa Barbara, CA, USA). Silicon nitride tips on V‐shaped gold‐coated cantilevers were used (0.01 N/m, MLCT; Veeco, Mannheim, Germany). Imaging was performed at ambient temperature with forces less than 1 nN at one to three scan lines per second (1–3 Hz) with a resolution of 512 × 512 pixels. For texture analysis, subcellular scan areas of 20 × 20 µm^2^ were recorded. For a larger overview, images of 70 × 70 µm^2^ were recorded. Topographical data of the corneocyte surfaces were analysed with the nAnostic™ method, by the use of custom‐built, proprietary algorithms (Serend‐ip, Münster, Germany). The method evaluates each nanostructure protruding from the mean surface level, referred to as circular nanosize objects (CNOs). These are then automatically filtered according to their size and shape; in the present study, only structures of positive local deviational volume smaller than 500 nm in height and with an area of <1 µm^2^ are considered. The DTI counts these features for an area of 20 × 20 µm^2^ of cell surface per image 9. For MCI/MI and SLS, SEM was performed on the tape strips from 1 person. Fragments of D‐Squame tapes were observed at a partial vacuum (0.133 kPa) without prior preparation of the samples (native state). Images of the removed corneocyte layers were recorded at 15 kV with the secondary electron detector of a Quanta 250 FEI scanning electron microscope.

### Statistics

Data analysis was performed with graphpad prism® version 6.07 (GraphPad Software, La Jolla, CA, USA). Comparison of the NMF levels between different skin sites (allergen/irritant/unpatched) and their corresponding pet. controls in the same individual was performed with a paired two‐tailed *t*‐test. Comparison of the activity of BH, C‐1 and plasmin between allergens and their pet. controls was performed with the Wilcoxon signed rank test if the distribution of data deviated from normal distribution as tested by the Shapiro–Wilk test, and with a two‐tailed *t*‐test when the data were normally distributed. The Spearman correlation coefficient was used to correlate the individual activities of the proteases with NMF levels. The Pearson correlation coefficient was used to correlate the individual patch test results (1+, 2+, and 3+) with NMF levels. One‐way anova followed by Dunnett's multiple comparison *post hoc* test was applied to the differences in DTI between SLS and individual allergens, pet., and unpatched skin sites. Data are shown as the mean value and standard deviation (SD) when distributed normally, and as median with interquartile range when non‐normally distributed.

## Results

### Clinical response

**Figure 1:**
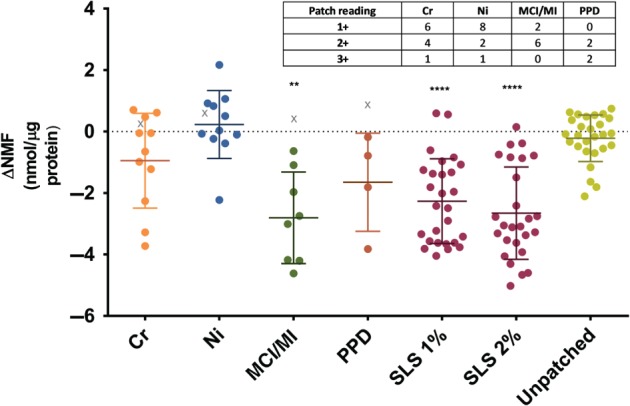
The difference in the natural moisturizing factor levels (ΔNMF) between the skin sites tested with potassium dichromate (Cr), nickel sulfate (Ni), methylchloroisothiazolinone (MCI)/methylisothiazolinone (MI) or p‐phenylenediamine (PPD), sodium lauryl sulfate (SLS), unpatched skin and their corresponding pet. controls on day 3. The individual patch test readings for each allergen are inserted as a table. As some patients had positive reactions to two allergens, the number of reactions (n = 34) is greater than the number of patients (n = 27). The ΔNMF of a positive reaction is indicated by a circle symbol, and that of a non‐responder is indicated by a ‘X’ symbol. Non‐responders were excluded from data analysis. The results are shown as mean of all subjects and standard deviation. The data of allergens, SLS and unpatched skin were compared with those of their corresponding pet. patches by use of a paired, two‐tailed t‐test. ^**^ p < 0.01; ^****^ p < 0.0001.

We included 27 patients (24 females), with an average age of 49.3 years (SD 12.6). A total of 34 positive 1+ or 2+, 3+ reactions to the investigated allergens were observed on D3: 11 for Ni, 11 for Cr, eight for MCI/MI, and four for PPD. Eleven patients had two positive reactions. Although selection was based on 1+ or 2+ reactions, several patients in the present study had 3+ reactions (one for Ni and Cr, and two for PPD). The clinical scores per allergen per patient are shown in Fig. 1

Four patients, 1 for each allergen, did not show a positive reaction to the allergen to which they had had a positive reaction in the past. Furthermore, 1 patient had a severe reaction to PPD, and the tape strip samples could not be obtained. These patients (n = 5) were excluded from the data analysis.

### NMF

Figure 1 shows the difference in the NMF levels (ΔNMF) between the allergens, SLS, unpatched sites, and their corresponding controls (pet.) on D3. A significant difference from the corresponding controls was observed for SLS (1% and 2%) and MCI/MI. The smallest effect was observed for Ni, for which none of the patients had ΔNMF lower than the median response after MCI/MI and SLS (Fig. 1). Although the difference with respect to the pet. control did not reach statistical significance for other allergens, several patients had negative ΔNMF values. For example, 3 patients for Ni and 1 for Cr and PPD showed NMF level decreases similar to the average decrease observed after MCI/MI and SLS (Fig. 1). Interestingly, those 5 patients had strong patch test reactions (3+ for Ni and Cr, and 2+ for PPD). To further explore possible associations between patch test readings and changes in NMF levels, we compared ΔNMF and patch test readings. The Pearson correlation coefficient amounted to −0.64 (*p* < 0.001), indicating a significant negative association between patch test reactions and decrease in NMF levels. The NMF levels after D2, determined in a limited number of patients, showed the same trend (data not shown). In each allergen group, 1 patient had no reaction to the allergen (denoted in Fig. 1. by an X symbol). As is evident from Fig. 1, the ΔNMF values in these subjects were close to those for the pet. control. There was no significant difference in the NMF levels between skin sites where no patches were applied and sites with pet.

### Corneocyte surface morphology

**Figure 2:**
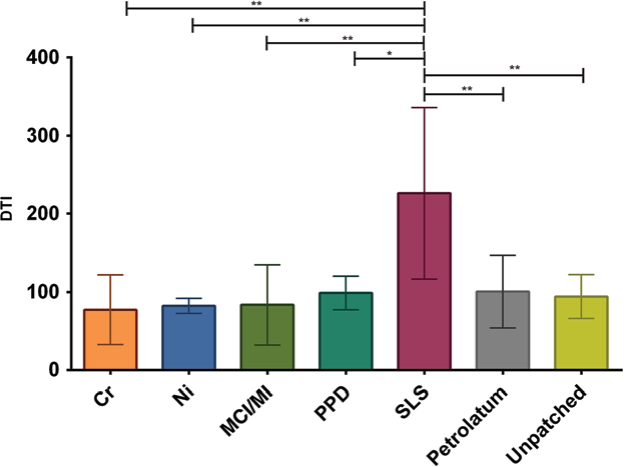
The Dermal Texture Index (DTI; number of circular nanosize objects per 20‐µm^2^ area) measured in the stratum corneum collected on day 3. The results are averaged for all subjects, and are shown as mean values and standard deviation. The number of tested sites per group differed: Cr, n = 3; Ni, n = 5; methylchloroisothiazolinone (MCI)/methylisothiazolinone (MI), n = 4; p‐phenylenediamine (PPD), n = 3; sodium lauryl sulfate (SLS), n = 6; pet., n = 7; and unpatched, n = 4. The DTI values of allergens/pet./unpatched skin were compared with those of the SLS group; asterisks indicate level of significance. ^**^ p < 0.01 (one‐way anova followed by Dunnett's multiple comparisons test).

**Figure 3:**
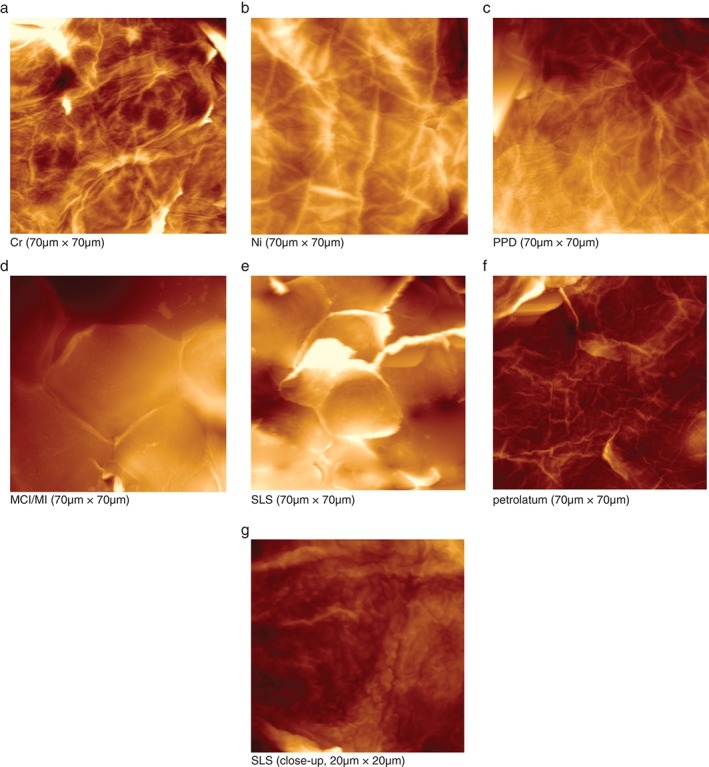
(a–g) Atomic force microscopy images from stratum corneum samples collected on day 3. (a) Chromium. (b) Nickel. (c) p‐Phenylenediamine. (d) Methylchloroisothiazolinone (MCI)/methylisothiazolinone (MI). (e) Sodium lauryl sulfate (SLS). (f) Pet. (g) SLS, close‐up. Images are three‐dimensional representations; the brightness corresponds to the height of the imaged structures. At the microscale (70 × 70 µm^2^), distinct morphological changes are seen for MCI/MI and SLS. On a close‐up view of an SLS sample (20 × 20 µm^2^) (g), circular nanosize objects can be distinguished on the corneocyte surface.

**Figure 4:**
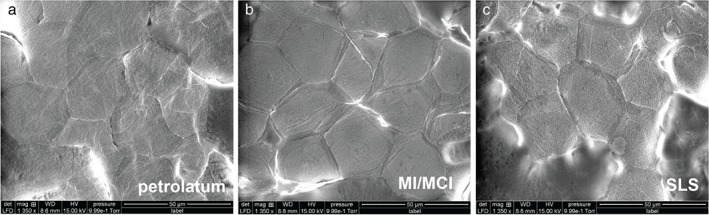
Scanning electron microscopy images of corneocytes on day 3 after application of pet. (a), methylchloroisothiazolinone (MCI)/methylisothiazolinone (MI) (b) and sodium lauryl sulfate (SLS) at ×1350 magnification. Note a loose lateral association between the cells for the MCI/MI and the SLS test sites. Furthermore, circular nanosize objects can be distinguished on the corneocyte surface of the SLS test site.

The DTI values were determined from the AFM images of a 20 × 20‐µm^2^ area. As shown in Fig. 2, among all investigated compounds, only SLS led to a significant rise in the DTI, indicating increased numbers of CNOs, which can clearly be seen from Fig. 3g, representing a more detailed 20 × 20‐µm^2^ image of an SLS‐tested skin site. The CNOs in the SLS image were also shown by SEM (Fig. 4). Larger overview AFM images (70 × 70 µm^2^) of the corneocytes from the skin sites tested with Cr, Ni, PPD, MCI/MI, SLS and pet. are shown in Fig. 3a–f. The images show that, at a microscopic level, the results for Cr, Ni and PPD resemble those for pet. MCI/MI differed, in that it caused distinct alterations in the structure; corneocytes were hexagonal‐shaped and had pronounced cell borders (Fig. 3d). The surfaces were smoother, with a loss of corneocyte surface microtexture. This was also confirmed by SEM images showing loose lateral associations between the cells from the MCI/MI‐treated skin sites (Fig. 4). As indicated in Fig. 2, these microscopic alterations did not lead to an increase in cell surface CNOs; the average DTI value from MCI/MI samples was similar to that for other allergens and pet.

### Activities of stratum corneum BH, C‐1, and plasmin

**Figure 5:**
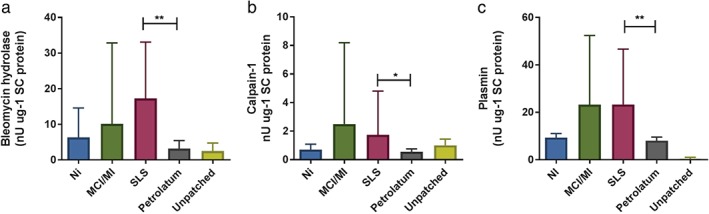
Activities of bleomycin hydrolase (a), calpain‐1 (b) and plasmin (c) in the stratum corneum samples of the skin sites tested with Ni (n = 4), methylchloroisothiazolinone (MCI)/methylisothiazolinone (MI) (n = 4), sodium lauryl sulfate (SLS) (n = 8) and their corresponding pet. controls (n = 8) and unpatched test sites (n = 8). Data for bleomycin hydrolase are shown as median with interquartile range; those for calpain‐1 (b) and plasmin (c) are shown as mean ± standard deviation. ^*^ p < 0.05, ^**^ p < 0.01; (a) Wilcoxon signed rank test; (b, c), paired two‐sided t‐test).

To explore whether allergens and SLS affect the activity of proteases that are involved in the degradation of NMF, we determined the activities of BH and C‐1 in a limited number of samples. Furthermore, we included plasmin as an indicator of skin barrier damage. The activities of BH, C‐1 and plasmin (Fig. 5a–c) were significantly higher in SLS‐treated skin than in the corresponding pet. controls (BH, *p* < 0.01; C‐1, *p* < 0.05; and plasmin, *p* < 0.01). The allergens did not produce significant differences from the pet. controls, although MCI/MI showed a trend of increasing values for all three proteases (each *p* = 0.13). The activities of all three proteases were negatively correlated with corresponding NMF levels. The respective Spearman correlation coefficients for BH, C‐1 and plasmin amounted to −0.52 (*p* < 0.01), −0.47 (*p* < 0.01), and −0.58 (*p* < 0.001).

## Discussion

In the present study, we observed different effects of contact allergens and SLS on relevant properties of the epidermal barrier: the stratum corneum NMF levels, corneocyte surface morphology, and stratum corneum protease activities. NMF levels have previously been used as a skin barrier biomarker in AD and ICD. To the best of our knowledge, this is the first time that NMF levels have been investigated in ACD 27, 28. Recent experimental studies showed that skin irritants with different physicochemical properties, such as SLS, NaOH, fruit acids and aliphatic alcohols, significantly decrease the stratum corneum NMF levels 28, 29. This is in accordance with the findings from the present study, which show a significant reduction in NMF levels after exposure to 1% and 2% SLS. SLS may potentially affect NMF levels in different ways. As an alkaline compound, SLS may lead to an increase in the stratum corneum pH, which might affect the activity of stratum corneum proteases, including those involved in filaggrin degradation into NMF components. To explore this possibility, we measured, in a limited number of samples, the activity of the stratum corneum proteases BH and C‐1, both of which known to be involved in breaking down filaggrin protein 30. The results suggest that it is unlikely that the decrease in NMF levels after SLS and MCI/MI treatment is caused by reduced activity of these enzymes, as their activities showed an opposite trend; protease activities were increased after SLS treatment, and an increasing trend was observed for MCI/MI (*p* = 0.07). The activities of these proteases were negatively correlated with NMF levels, so the increased activity might be a feedback reaction to the reduced NMF levels. SLS is known to denature proteins of the cornified envelope, which may lead to the leakage of NMF components from the corneocytes 31. This could also occur for the proteases, causing better extraction from the corneocytes and/or intercellular lipids. As the cornified envelope acts as an attachment point for the intercellular lipids, disruption of the cornified envelope additionally affects skin barrier function 32, 33. Further evidence that the reduction in NMF levels may be caused by skin barrier damage is provided by increased plasmin activity following SLS treatment (*p* < 0.01), which indicates a damaged skin barrier 18. A trend of increasing plasmin activity was also observed after MCI/MI treatment (*p* = 0.13).

Among the tested allergens, only MCI/MI caused a significant reduction in NMF levels. It is not likely that the NMF decrease after MCI/MI treatment is attributable to downregulation of (pro)filaggrin, as the stratum corneum samples originate from the upper part of the stratum corneum (approximately to the upper third of the stratum corneum depth). The deeper stratum corneum layers containing potentially downregulated expression of filaggrin would require a further 14 days to reach the more superficial part of the stratum corneum from which the samples originated 34. As NMF is mainly located within the corneocyte, where filaggrin degradation occurs, it might be speculated that MCI/MI, like SLS, damages the cornified envelope, resulting in leakage of NMF. MCI (the most abundant component in the 3:1 MCI/MI mixture) is a small lipophilic compound with favourable physicochemical properties for percutaneous penetration across the membrane (*K*_ow_ = 2.5; MW 111). It has been shown that MCI has corrosive properties and is retained in the epidermis, probably because of binding to the epidermal proteins 35, 36. In the present study, MCI/MI treatment resulted in dramatic changes in the microscale corneocyte structure, characterized by the smoother corneocyte surfaces, the hexagonal shape, pronounced cell borders, and the absence of apparent fibrous structures, that were distinctly different from the effects of the other three allergens. As recently reviewed by Weidinger and Novak, a compromised barrier may facilitate sensitization and increased penetration of contact allergens 37. In murine studies, the irritant effect of an allergen was shown to determine the strength of the contact hypersensitivity response 11. The decrease in NMF levels observed in this study might at least partly have been caused by irritant characteristics of MCI/MI, and this is perhaps an explanation for its high allergenic potency. Although Ni and Cr did not lead to significant changes in NMF levels, individuals with the highest clinical scoring showed the lowest NMF levels, suggesting that allergen‐induced inflammation and decreases in NMF levels are associated.

At the topographical scale, quantified by the number of CNOs (expressed as the DTI), MCI/MI did not differ from the other allergens, and SLS was the only substance showing increased DTI values. Increases in DTI have recently also been found for other skin irritants, such as NaOH and lactic acid (C. Riethmuller, et al. pers. comm. 2016), suggesting that an elevated DTI is characteristic of skin irritation. The mechanisms that underlie the development of CNOs are not yet clear. In another study by Riethmuller et al., AD patients with compound heterozygote or homozygote loss‐of‐function mutations in the filaggrin gene were shown to have increased numbers of CNOs 9. These patients lack filaggrin, which aggregates keratin filaments within the corneocyte and is also present in the cornified envelope. It might be suggested that, owing to the lack of filaggrin, the cornified envelope is more fragile and becomes more prone to structural changes resulting from osmotic pressure within the corneocytes caused by reduced NMF levels. Interestingly, this study shows that allergen‐induced inflammation does not result in the formation of CNOs, regardless of the low NMF levels, indicating that their formation is multifactorial.

If the finding that the DTI does not change in ACD, as we show for four clinically relevant allergens, can be generalized to other allergens, the DTI might aid in differentiating ACD from ICD. However, it has to be noted that, in the occupational setting, mixed exposure to allergens and skin irritants is common, so results might be less clear than in this controlled study. Moreover, many allergens have irritant properties, so an increased DTI does not necessarily exclude ACD. Nevertheless, the investigated parameters can provide more insights in the aetiology of ICD and AD and the intrinsic irritant properties of contact allergens, which might support more targeted prevention in occupational settings.

## Conclusion

Skin barrier characteristics, for example NMF levels and the number of nanosize objects (DTI) on the corneocyte surface, are useful for studying the effects of skin irritants and contact sensitizers on the epidermis. In contrast to the other allergens investigated, MCI/MI showed distinct effects on the skin barrier in terms of a significant decrease in NMF levels, similarly to SLS, and MCI/MI also had profound effects on corneocyte morphology; collectively, these findings suggest that skin barrier damage plays a role in the pathogenesis of MCI/MI contact allergy. The DTI seems to differentiate reactions to the tested allergens and to SLS, as the latter was the only agent that caused an increase in the DTI. Whether the effects on NMF levels and the DTI can be generalized to other skin irritants should be confirmed in further studies including irritants with different physicochemical properties.
